# Governing partnerships with technology companies as part of the COVID-19 response in Canada: A qualitative case study

**DOI:** 10.1371/journal.pdig.0000164

**Published:** 2022-12-14

**Authors:** Vinyas Harish, Thomas G. Samson, Lori Diemert, Ashleigh Tuite, Muhammad Mamdani, Kamran Khan, Anita McGahan, James A. Shaw, Sunit Das, Laura C. Rosella

**Affiliations:** 1 MD/PhD Program, Temerty Faculty of Medicine, University of Toronto, Toronto, Canada; 2 Dalla Lana School of Public Health, University of Toronto, Toronto, Canada; 3 Temerty Centre for Artificial Intelligence Research and Education in Medicine (T-CAIREM), Temerty Faculty of Medicine, University of Toronto, Toronto, Canada; 4 Vector Institute for Artificial Intelligence, Toronto, Canada; 5 Schwartz Reisman Institute for Technology and Society, Toronto, Canada; 6 Ethics of AI Lab, Centre for Ethics, University of Toronto, Toronto, Canada; 7 Li Ka Shing Knowledge Institute, Unity Health Toronto, Toronto, Canada; 8 Division of Infectious Diseases, Department of Medicine, Temerty Faculty of Medicine, University of Toronto, Toronto, Canada; 9 Rotman School of Management, University of Toronto, Toronto, Canada; 10 Munk School of Global Affairs and Public Policy, University of Toronto, Toronto, Canada; 11 Joint Centre for Bioethics, Dalla Lana School of Public Health, University of Toronto, Toronto, Canada; 12 Department of Physical Therapy, Temerty Faculty of Medicine, University of Toronto, Toronto, Canada; 13 Institute for Health Systems Solutions and Virtual Care, Women’s College Hospital, Toronto, Canada; 14 Division of Neurosurgery, Department of Surgery, Temerty Faculty of Medicine, Toronto, Canada; 15 Institute for Better Health, Trillium Health Partners, Mississauga, Canada; University of British Columbia, CANADA

## Abstract

Cross-sector partnerships are vital for maintaining resilient health systems; however, few studies have sought to empirically assess the barriers and enablers of effective and responsible partnerships during public health emergencies. Through a qualitative, multiple case study, we analyzed 210 documents and conducted 26 interviews with stakeholders in three real-world partnerships between Canadian health organizations and private technology startups during the COVID-19 pandemic. The three partnerships involved: 1) deploying a virtual care platform to care for COVID-19 patients at one hospital, 2) deploying a secure messaging platform for physicians at another hospital, and 3) using data science to support a public health organization. Our results demonstrate that a public health emergency created time and resource pressures throughout a partnership. Given these constraints, early and sustained alignment on the core problem was critical for success. Moreover, governance processes designed for normal operations, such as procurement, were triaged and streamlined. Social learning, or the process of learning from observing others, offset some time and resource pressures. Social learning took many forms ranging from informal conversations between individuals at peer organisations (e.g., hospital chief information officers) to standing meetings at the local university’s city-wide COVID-19 response table. We also found that startups’ flexibility and understanding of the local context enabled them to play a highly valuable role in emergency response. However, pandemic fueled “hypergrowth” created risks for startups, such as introducing opportunities for deviation away from their core value proposition. Finally, we found each partnership navigated intense workloads, burnout, and personnel turnover through the pandemic. Strong partnerships required healthy, motivated teams. Visibility into and engagement in partnership governance, belief in partnership impact, and strong emotional intelligence in managers promoted team well-being. Taken together, these findings can help to bridge the theory-to-practice gap and guide effective cross-sector partnerships during public health emergencies.

## Author summary

Digital technologies have played a unprecedented role in emergency response during the COVID-19 pandemic. Partnerships between private sector technology companies and health institutions, such as hospitals and public health organizations, are often necessary to develop and deploy digital technologies. Here, we sought to understand the barriers and enablers to forming effective and responsible cross-sector partnerships during a public health emergency—with a focus on the role of startups. The firms we studied triaged and streamlined various governance processes (e.g., stakeholder assessment, contracting, procurement) in response to COVID-19’s time and resource pressures. Informants articulated the paramount importance of achieving early and sustained alignment on the core problem to be solved and how governance structures (e.g., connections between team members at different levels of seniority) facilitated this alignment. Flexibility and deep knowledge of the local operating context provided startups with an immense competitive advantage. Key areas for improving partnerships include: knowledge translation to promote the uptake of governance frameworks among decision-makers, accelerating public sector procurement processes that are too long and complex to be financially viable for startups, and developing pragmatic, public engagement strategies to build trust and promote social acceptability.

## Introduction

COVID-19 has upended health systems, economies, and daily life around the globe. Given the immense burden the pandemic has placed on health systems, there has been a resurgence in the discussion of health systems resilience among practitioners and researchers [1]. Health systems resilience reflects preparedness for an emergency as well as the capacity in the system to respond effectively to emergencies while maintaining core functions [2]. Health emergencies such as pandemics have broad impacts across society and thus a key principle that characterizes resilient health systems is the capacity to coordinate actions across the public, private, and non-profit sectors [2].

Past public health crises such as pandemic H1N1 and the 2014–2016 Ebola epidemic have generated calls to action around fostering effective cross-sector partnerships [3–5]. The COVID-19 crisis is unique due to the previously unprecedented role of digital technologies, and thus private technology companies, in pandemic response [6]. From digital contact tracing (DCT), to ledgers organizing vaccine administration, to machine learning-based risk prediction models of severe clinical outcomes—medical and public health organizations have sought to leverage digital technologies to support pandemic response [7–9]. However, health organizations traditionally do not have the skillset to develop and deploy such technologies in-house, especially in the context of a rapidly evolving public health emergency [10]. Therefore, partnerships with private technology companies, both large and small, have become increasingly common. The governance arrangements underlying these collaborations are essential for social license as well as the likelihood and success of technology development and uptake [11].

The concept of social license, or a social license to operate, is a community’s perceptions of the acceptability of a company and its local operations [12]. It refers to the informal permissions granted to institutions such as governments or corporations to carry out a particular set of activities [11]. Building social license encapsulates legitimacy, credibility, and trust between a given organization, stakeholders within and outside of said organization, and the general public [12,13]. As such, civil society may have demands or expectations that surpass those imposed by legal regulations [14]. One area particularly relevant to partnerships between health organizations and private-technology companies is data privacy and security. Personal information in Canada is protected by different regulations depending on the handling entity: the Privacy Act for the federal government [15], the Personal Information Protection and Electronic Documents Act (PIPEDA) for private sector organizations [16], and provincial health legislation for hospitals and other similar institutions that serve as custodians of personal health information. In Ontario, Canada’s largest province, the data legislation for hospitals, long-term care homes, and pharmacies is titled “the Personal Health Information Act” (PHIPA) [17]. Even if a partnership adheres to these regulations, it is possible that it may not obtain social license due to a wide range of governance factors such as a lack of transparency, lack of public engagement, and data sharing beyond what is perceived necessary [11].

While there is growing discourse around the ethics of collaborations between technology companies and health organizations [18], there is a dearth of research investigating this type of collaboration in-depth as it is deployed during a public emergency. During the COVID-19 pandemic, much attention has been placed on DCT, but DCT is only one specific type of technology deployment within a broader taxonomy of digital health tools [19–21]. Finally, much of the literature in this area focuses on major multinational companies, such as Google DeepMind and IBM [22,23]. While these “Big Tech” companies have formed over thirty partnerships with hospitals and health systems globally, they are not the only players in this space [24]. Funding for digital health startups has been growing year-over-year, with US startups raising almost $30B USD and European startups raising $8.1B USD in 2021 [25,26]. Throughout the pandemic, health organizations and startups around the world have formed partnerships across clinical, operational, and population health domains [27–29]. Compared to Big Tech, startups face the “liability of newness” and “liability of smallness” [30]. Since startups are often small and resource-scarce, they are can be vulnerable to financial pressures, human resource shifts, and external shocks [31]. They may also lack established business models and market legitimacy [30–32]. Forming partnerships is vital to the long-term survival and sustainability of startups [33,34]. From the perspective of health systems partners, the fundamental characteristics of “smallness” and “newness” can also make startups exceedingly valuable as partners. Research from the 2008–2009 financial crisis has found that small firms in the UK and New Zealand demonstrated high levels of adaptability and flexibility [35]. Given their size, local startups may be more accessible to health organizations and logistically easier to partner with. Both smallness and newness also require decision-makers in startups to attend closely to their customers and local market context [36]. Accessibility and understanding of local context can also be magnified by mutual engagement within the local innovation ecosystem [37]. Although partnerships with startups often do not create as much public attention, and perhaps criticism or outrage, as partnerships with Big Tech companies, stakeholders cannot assume partnernships with startups will automatically have social license [38–40]. Ultimately, how best to govern these partnerships in a manner that promotes social license remains an open question that warrants detailed study [41].

Using a qualitative, multiple case study design, we sought to study the governance arrangements between Canadian health organizations and private technology startups during the COVID-19 pandemic. Our objective was to systematically apply a framework of adaptive governance in healthcare to describe and analyze the barriers and enablers of deploying digital technologies as part of the pandemic response. The results of this work are vital to help bridge the theory-to-practice gap and to guide effective and responsible cross-sector partnerships during public health emergencies [42].

## Methods

This qualitative study was conducted through use of a multiple case study design [43]. Using a case study design allows researchers to illuminate why a decision–or set of decisions–were taken, how they were implemented, and with what results. When describing case-study research in his popular methodological manual, Robert K. Yin puts forth that a case study “relies on multiple sources of evidence, with data needing to converge in a triangulating fashion” [43]. Examples of common evidence sources researchers select to conduct in-depth study of a phenomenon in its real-world context include: documentation, archival records, interviews, direct observations, participant-observation, and physical artifacts [43]. We selected the complementary methods of a document review as well as interviews with key informants. The purpose of the document review was to provide context into the need for study partnerships, provide a starting point for identifying potential key informants, and gain an understanding into key events and outcomes of the partnerships. Key informant interviews were conducted to explore a range of topics from the drawn upon theoretical frameworks (i.e., Vayena and Blasimme’s framework of systemic oversight, Gasser’s navigation aid for the ethical use of digital public health tools) that we believed would not likely be captured in documents. Key informant interviews can elucidate why and how certain decisions were made—that is provide critical perspectives into partnership governance. Interviews also allow for researchers to challenge their participants by probing on what they thought could have gone differently or be improved upon. Ultimately, we believed that document review and the key informant interviews would provide us with complementary and synergistic findings and contribute to a robust case study. We explain the methodological basis for how we conducted both below.

Our cases were sampled from three separate public/private collaborations to deploy digital technologies as part of the COVID-19 response. These collaborations were broken down into three categories of technologies as outlined by Shaw *et al*. (2019): clinical, operational, and epidemiological [44]. While there are other taxonomies that have been applied to digital technologies in the context of the pandemic [20,21], these taxonomies and their subcategories are not mutually exclusive. Each of the technology companies were startups.

Technology company A and health organization B partnered to deploy a virtual care platform for staff at long-term care homes to consult physicians regarding the status of COVID-19 patients without having to send them to an emergency room. This virtual care platform also facilitated COVID-19 testing for patients within health organization’s catchment area. Shaw *et al*. (2019) defined “clinical applications” as those pertaining to the tasks of assessing, evaluating, and intervening on health issues [44]. Since these applications are directly related to the task of patient assessment and evaluation, we selected this partnership as our “clinical” collaboration.

Technology company C and health organization D partnered to deploy a secure messaging platform for physicians. Physicians used this platform, in part, to coordinate the care of COVID-19 patients. Physicians would receive notifications on this platform when tests returned positive with SARS-CoV-2, use the platform to request consults with other services, and serve as a digital pager when on call. As per the definition proposed by Shaw *et al*. (2019), these tasks are ancillary to, but essential for delivering clinical services [44]. Therefore, we selected this partnership as our “operational” collaboration.

Technology company E partnered with health organization F to provide data science consulting regarding the management and spread of COVID-19 at the population-level. Part of this consulting involved leveraging novel, digital data streams. As such, we selected this partnership as our “epidemiological” collaboration of study in line with the definition proposed by Shaw *et al*. (2019) [44].

The methods were performed in accordance with relevant guidelines and regulations and approved by the University of Toronto’s Health Sciences Research Ethics Board (REB #26448).

### Theoretical framework

Since we are studying the governance of collaborations between Canadian public health/health care organizations and private technology companies, we have drawn from a governance framework to inform the methodologies and analysis of our study. Specifically, we used Vayena and Blassime’s framework of systemic oversight [45–47].

Systemic oversight draws upon the extensive literature on adaptive governance, originally developed for application in the environmental sciences. Adaptive governance is a means of coordinating resource management within uncertainty-laden and rapidly changing socio-ecological systems [48,49]. Vayena and Blassime argue that health data, due to its sources, capabilities, and stakeholders, can be characterized as an “ecosystem”, presenting significant implications for its management [45]. The framework is aimed at promoting responsible innovation across biomedical research, patient care, and public health with three aims: 1) to draw attention to ethically controversial areas to address risks, 2) to direct innovation towards outcomes beneficial for society, and 3) to promote public trust and accountability [46]. In keeping with these objectives, systemic oversight: 1) leverages adaptive and flexible mechanisms to cope with uncertainty, 2) is both inclusive and reflexive to engage a wide range of stakeholders and consider how they may be impacted by a given solution, and 3) monitors for and is responsive to emerging risks and harms [45–47].

There are numerous conceptual strengths of this framework for our study. First, it recognizes the ecosystemic and highly interconnected nature of both the digital technologies and the institutions involved in COVID-19 response. Second, it is appropriate for understanding how organizations respond to the COVID-19 pandemic, which is inherently a situation in which actors must act under tremendous uncertainty, time pressures, and constant change (e.g., in the epidemiological and clinical dynamics of the disease, spillover impacts to other aspects of society such as supply chains, etc.). Adaptive governance has been used in similar areas such as managing and mitigating risk due to natural disasters [50]. Third, adaptive governance also aptly relates to the notion of resiliency as both draw from the environmental sciences [48]. Finally, the use of this framework lends itself towards a hypothesis that is grounded in what has been seen in adjacent literature: when the structure of an ecosystem is challenged by crisis, then the mechanisms of governance that were designed optimally to support the ecosystem are at the heart of the crisis and must adapt in ways for which they were not designed.

We opted to employ Blasimme and Vayena’s framework of systemic oversight over other frameworks such as Char’s pipeline [51] or Leslie’s process-based governance framework for AI workflows [52] due to its complementarity and specificity with this study (i.e., we are focused on the governance of a health response to a crisis rather than the ethics surrounding a response). We also focus on the use of digital technologies to meet this response rather than artificial intelligence tools, strictly speaking. We also drew from Gasser *et al*.’s navigation aid to guide our data collection through semi-structured interviews with key informants [20]. We structured our interviews in accordance with the aid’s conceptual phases of developing and deploying digital technologies: 1) Preparation, 2) Planning, 3) Risk Assessment, 4) Development, 5) Deployment and Evaluation.

### Document review

Document analysis included documents that provided details on the structure and nature of the sampled partnerships. Documents were included if they included details of the partnership, leadership perspectives, outcome measures, regulatory landscape, or product features. The search for these documents was done through multiple Google searches and manual searches in sampled organization webpages by following links. This process was done independently by two individuals (VH, TS) while keeping track of search terms (Appendix A in S1 Text). Searching and data abstraction was done by two independent reviewers (VH and TS), with conflicts resolved by discussion. We performed two rounds of document review (summer 2021, winter 2022) to account for new updates regarding the partnerships or organizations involved during the study period.

### Key informant interviews

An initial list of key informants was first identified through the document analysis. We then used snowball sampling to identify additional participants at the end of each interview. Participants were recruited by email and were sent a standardized consent form (Appendix B in S1 Text). We sought to recruit participants in the roles of organizational leadership, end-users, and project/product managers, among others. To promote participants speaking freely, we ensured that their identities would be kept anonymous throughout the course of the study and requested that managers not ask if employees participated in the study.

The semi-structured interview guide (Appendix C in S1 Text) served to explore a range of topics from Vayena and Blasimme’s framework of systemic oversight as well as Gasser and colleagues’ navigation aid for the ethical use of digital public health tools [20,45–47].

Using this semi-structured interview guide, we conducted six pilot interviews prior to interviewing key informants to refine the content of the interview guide as well as the length and structure of the interview. We piloted with senior members of the research team and colleagues in similar roles to our key informants (e.g., clinicians, data scientists, etc.), and thus, we did not include the pilot interviews in the analysis.

The consent form was emailed to participants ahead of time, and interviews were conducted over internet-based audio/video chat on Zoom. Interviews were an hour on average and consenting participants had the option to be audio/video recorded. Interviews were then transcribed verbatim using Zoom’s built-in, automated transcription feature and the adequacy of the transcriptions were assessed by VH and TS using recordings. Recordings were then deleted upon ensuring the adequacy of transcription.

### Coding and data analysis

To abstract and synthesize data from key informant interviews and publicly available documents, data coding was done iteratively and through the qualitative method of abductive analysis [53]. We selected abductive analysis to move between the data and our *a priori* theories. The goal of the abductive analysis is to generate novel theoretical insights that reframe empirical findings in contrast to existing theories. The transcribed interviews and document analysis were described using the coding structure-specific in Appendix D in S1 Text. Additional coding domains were developed as needed during the analysis, and the key themes were identified as nodes within the qualitative software package.

As mentioned above, the primary framework used to guide analysis was Blasimme and Vayena’s framework of adaptive governance through systemic oversight because it highlights a range of recommendations that promote responsible big data innovation across biomedical research, patient care, and public health. It also emphasizes the importance of directing attention to ethically controversial areas to address risks while also promoting public trust and accountability. Throughout the analysis, we also allowed thematic content to evolve independently of Blasimme and Vayena’s framework.

All coding was done in duplicate by VH and TS with conflicts resolved by discussion for each document and transcript until consensus was reached. Data collection, organization, and analysis was done using the NVivo qualitative software (version 12, QSR International, Melbourne, Australia). Finally, the data from each case study was gathered and synthesized into a single brief case report to facilitate cross-case synthesis. The results of the cross-case synthesis are presented here. Recruitment and data analysis were done in an iterative process between July 2021 and February 2022.

### Sample size adequacy

In keeping with case study methodology, our goal was not to speak to every single stakeholder in each case but rather to understand an issue, process, or phenomenon. We used information power as the criteria for stopping further recruitment in each case once we believed we had a clear and sufficient understanding of the phenomena in each case [54]. Due to our narrow aim (i.e., explore specific partnerships between private technology companies and public sector health organizations), specificity in experiences of our key informants (i.e., had direct involvement with partnerships during COVID-19), use of well-established theory (i.e., Blasimme and Vayena’s theory of adaptive governance), and richness of interview dialogue, we found that five to ten interviews was sufficient to gain an understanding of each case in combination with the documents obtained. We then applied these requirements across our three cases.

## Results

**Fig 1:**
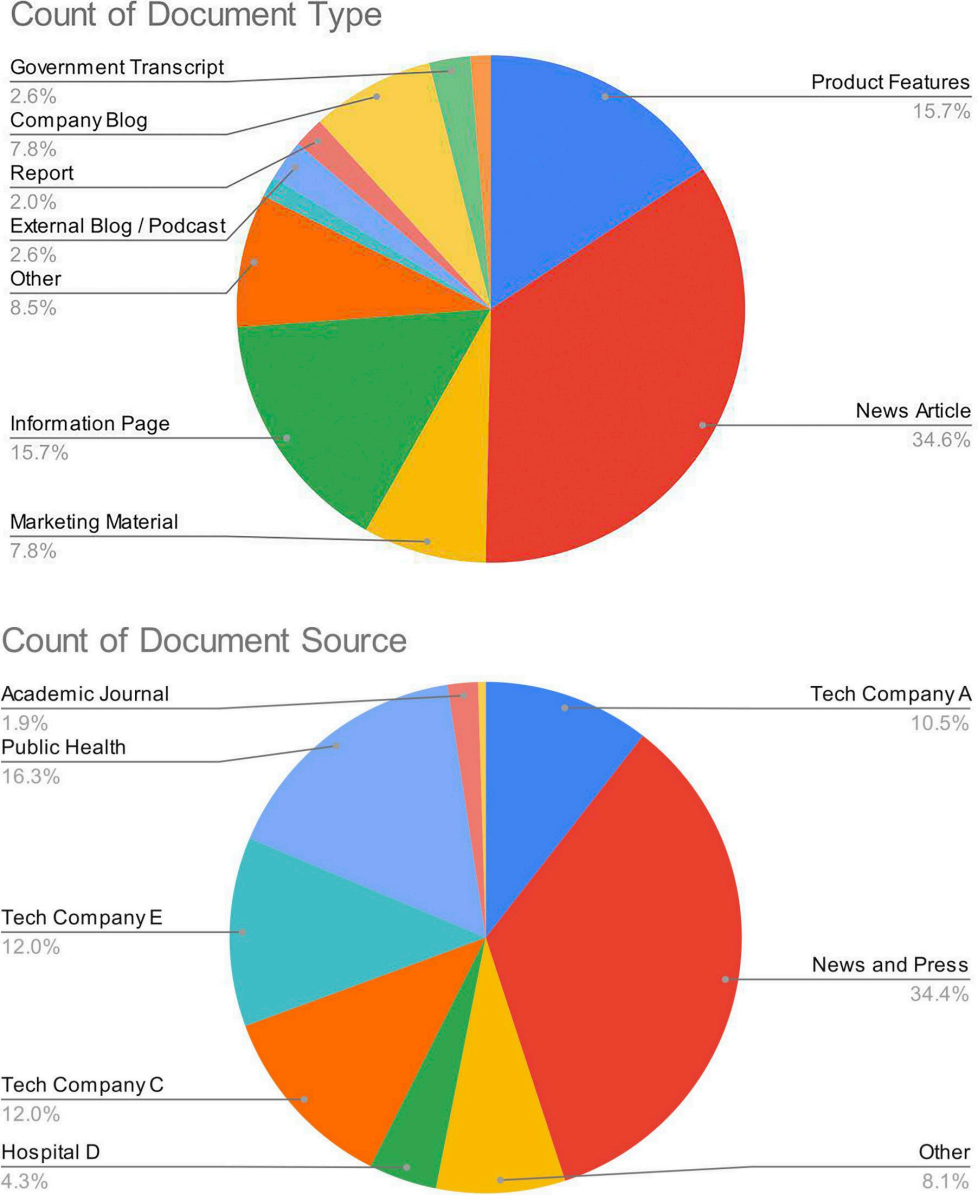
Summary of documents obtained. Pie charts of document type (A) and source (B) from document review, n = 210.

We obtained 210 publicly available documents and conducted 26 interviews on our three cases of study between June 2021 and February 2022. A breakdown on the sources and types of documents is presented in Fig 1. Most documents were news articles, marketing materials, and webpages (Fig 1A). Most documents were obtained from company websites (Fig 1B). In what follows, we describe several major themes that emerged over our iterative, abductive analysis of these documents and interviews in relation to our guiding theories. Despite the considerable pressures of the pandemic, we found that each partnership was able to enact key aspects of adaptive governance along the pipeline of solution development to deployment (Table 1). We describe how a health emergency created pressures for our cases and how these pressures were navigated below. We first begin with the time and resource pressures created by the pandemic (theme 1), and then discuss how those pressures demand alignment early in the partnership (theme 2) and the streamlining of governance processes throughout the partnership (theme 3). We then present findings regarding a particularly important governance process, social learning, in detail (theme 4). This is followed by an outline of the specific opportunities and risks for startups during a health emergency (theme 5). Finally, we end with how teams had to navigate all these pressures and remain high-functioning (theme 6).

**Table 1:** Mapping the six higher-order principles of adaptive governance by systemic oversight (Blasimme and Vayena, JMLE 2018) to key events or concepts in the three cases of study.

	Case of Study		
Adaptive Governance Principle	*Clinical*	*Operational*	*Epidemiological*
***Adaptivity / Flexibility*** *How do partners navigate the use of novel technologies broadly*, *especially in changing organizational contexts*?	- Pandemic motivated the Ontario government to create new physician billing codes for virtual care - Hospital B had previously done some diligence on tech company A and were able to move faster in collaboration since tech company was viewed as a trusted partner - Hospital B had an ongoing roll out of new electronic medical record, which changed workflows - Patients/families in LTCH were able to withdraw consent from virtual assessments	- Hospital D and tech company C had established partnership and had already completed pilot and began to roll out solution prior to pandemic - Pandemic accelerated scale-up of solution through hospital, key COVID-19 use cases were used to pilot new features/applications of solution - Tech company C provided additional flexibility in contracting to accommodate hospital’s community partners	- Tech company E beginning to organize around highly agile and cross-functional teams to break down silos between departments (e.g., finance, data science, product, etc.) - Tech company E had been exploring novel data streams to inform public health response for over two years prior to the pandemic, developing key internal mechanisms and safeguards - Tech company E regularly publishes in and seeks inspiration from scientific literature to guide products and consulting outputs with novel data streams, viewing peer review of methodologies to be an important gauge of quality - Public health organization F has been exploring how to modernize data science efforts and industry partnerships prior to pandemic - Public health organization F activated an emergency operations centre to centrally coordinate response related to COVID-19 - Public health organization F exploring novel methods of procurement (e.g., challenge-based procurement)
***Inclusiveness*** *What stakeholders are at the table throughout the partnership*?	- Patient and family advisory committee provided feedback on LTCH solution throughout deployment - Medical advisory committee provided feedback on LTCH solution throughout deployment	- Hospital D fostered high-quality, longitudinal engagement with a wide range of community partners - Tech company C spent hundreds of hours engaging with (e.g., interviewing, shadowing) hospital administrators, clinicians, etc. to understand workflows	- Public health organization F consulted privacy, legal, and ethics experts to inform efforts with novel data streams - Tech company E had an established working group as well as open door meetings to discuss how to navigate ethical challenges posed by novel data streams—including around ethical “red lines”
***Responsiveness*** *How do partners ensure that risks do not translate into real-world harms*?	- Hospital B had to navigate confusion from patients around if they would be charged for COVID-19 screening due to partnership with tech company A - Tech company A had to navigate massive increase in demand for services (e.g., onboarding new physicians, server upgrades, replying to customer support tickets)	- Tech company C was able to provide a high degree of customization in response to user needs (e.g., in product features, training materials, etc.) - Hospital D had to navigate potential liability with external community partners using their “instance” of the solution	- Tech company E had well established measures to control for privacy (e.g., spatiotemporal aggregation), security (e.g., two-factor authentication), and quality (e.g., review of outputs at multiple stages by managers) when users were working with novel data streams to generate consulting outputs - Tech company E created automated quality assurance bots to continuously ensure integrity of back-end data pipelines - Tech company E created streamlined workflows on Slack to respond to client issues as they appeared (e.g., who discovered the problem, what causes the problem, who is working on a fix, etc.)
***Reflexivity*** *Are partners aware of the risks posed by emerging technologies in their respective domains*?	- Hospital B Leadership recognized that older adults are medically complex, require care from multiple specialists at a given time, have additional cognitive/communication barriers and can rapidly deteriorate if transported to ED–providing “burning platform” for deployment during pandemic - Vulnerable populations (e.g., recent immigrants, refugees) may not have a health insurance card to register with; could leave the field blank - Vulnerable populations may not have devices or internet access to engage with virtual screening; could still arrive to get testing conventionally	- Hospital D leadership recognized that physicians and other health providers were already relying on non-secure and inefficient solutions for care coordination (e.g., Whatsapp, texting, etc.)–providing “burning platform” for accelerating deployment during pandemic - Hospital D leadership recognized that COVID-19 patients could deteriorate rapidly and that care coordination involves numerous providers/specialists (e.g., infectious diseases) when designing workflows	- Leadership across the partnership recognized the ethical and legal implications of working with novel data streams to inform public health response; sought to minimize the spatiotemporal granularity of these streams at all phases of analysis in keeping with ethical principles and legal responsibilities (e.g., data sharing agreements, contracts with vendors)
***Monitoring*** *How do partners detect and keep abreast of emerging risks*, *both in terms of outputs and overall partnership direction*?	- Ongoing meetings and alignment between C-Suite at hospital B and tech company A - Tech company A had standard practice of using a risk registry that would be updated through project life cycle - Patients could directly provide feedback into the virtual care platform, which was integrated into product roadmaps - Clearly measurable KPIs were developed in alignment with strategic objectives (e.g., reduce avoidable hospital transfers) - Tech company A recently completed provincial vendor verification, meeting standards for privacy, security, and interoperability	- Ongoing meetings and alignment between C-Suite at hospital D and tech company C - Clearly measurable KPIs were developed where possible - Continuously evaluated expansion of features / workflows against core value proposition of solution - Tech company C continuously sought out feedback from diverse user groups to inform product roadmap and features Tech company C recently completed detailed security certification and displays key features on a web-based dashboard	- Ongoing meetings between C-Suite at tech company and high-level managers at public health organization F - Tech company E continuously sought out feedback from users to inform consulting outputs and product roadmap - Tech company E recently completed security audit and will be bringing on in-house security expertise to guide work with novel data streams

**Acronyms:** LTCH–Long-term care home; ED–Emergency department; KPI–Key performance indicators

### A health emergency ultimately creates tension with time and resources

COVID-19 has been all-consuming for public sector organizations, especially during surges when staff are pulled away from non-pandemic-related activities to maintain core functions.

*“… you’re dealing with a pandemic that is pulling every and all possible resources towards it like a big giant star.” (Case 1—Interview -Sales)*

The ever-changing nature of the pandemic also created an environment of uncertainty for the partnerships as the health organizations we studied had to respond on very short timelines to developments both in the dynamics of the pandemic itself (e.g., the emergence of new variants such as Delta) or in pandemic-related policies (e.g., changes in testing criteria). These immense demands either catalyzed partnerships with private technology companies to form *de novo* or for existing partnerships to expand into new areas in reaction to evolving needs, such as keeping track of which patients were COVID-positive in the hospital or who received COVID-19 vaccinations in the community.

“*So one of the challenges was that patients who were positive were a needle in the haystack amidst all the negative test… [the solution we built] ensured that everyone was on the same page within an hour of the resulting positive*.*” (Case 2—Webinar—Chief Medical Information Officer)*

These pressures were felt across the different stages of the partnership (Table 2). At the beginning of the partnership, many participants felt that the current procurement regulations for public organizations, including hospitals, were not sufficiently agile in response to the time pressures of the pandemic.

**Table 2:** Mapping the barriers and enablers identified in our three cases of study to the recommendations in Gasser’ *et al*.’s navigation aid for the ethical development and use of digital tools during the COVID-19 pandemic. (Gasser *et al*., Lancet Digital Health 2020).

Recommendations by Stage of Navigation Aid	Enablers	Barriers	Quotes
***Preparation*** *Assemble the right team*, *establish guiding principles including ethical principles*	- Supportive and long-standing relationships between partner organizations - Knowledge of key stakeholders, especially by the health organization - Common objectives - Streamlined procurement processes - Understanding the core problem that needs to be solved - Patience, empathy, and other people skills - Social learning - Pragmatic public engagement	- Lengthy procurement process - Disengagement among end-users/leadership - Limited time and resources - Unfamiliarity with frameworks or best practices that help stakeholders identify and operationalize ethical principles	“Usually what I like to do is first and foremost, establish the project governance so I need to know right … out of the gate, who are the key or core project members on the partner client side. So, once I knew that and we’ve already identified those folks on the medical side. Then I usually put in standing meetings into calendars where this is where the core project team meets.” (Case 1 –Interview–Implementation Manager) “…same thing with with kind of the social license, and the trust of people, if you’re doing it in the middle of an emergency you’re you’re doing it too late. So that trust needs to be built … part of the work that we’re spinning up is to actually set up a community of practice or a community around public health data where we’ve got privacy advocates and where we’ve got you know technologists and where we’ve got people who care passionately about public health and about data together and where we can explore these things” (Case 3 –Interview–Chief Data Officer)
***Planning*** *Distinguish tools from purpose*, *avoid lock-in and path dependency*	- Ability to remain adaptable as problem/context changes - Engaging key stakeholders internally and externally - Effective kick-off meetings - Clear and explicit list of expectations - Patience, empathy, and other people skills - Social learning	- Lack of end user buy-in - Bureaucracy with larger organizations - Lack of transparency with partner organization - Dissipating excitement and engagement among leadership and champions - Limited time and resources	“[Kick off meetings are] … just more collaborative. People will ask questions people will confirm things people will bring up ideas during the kickoff call and it just, it makes for a much better deployment I think when you have… really engaged project leads.” (Case 2 –Interview–Customer Success) “So, and I say this because … a lot of times companies don’t want to admit that maybe they made a mistake, or … they’re really really busy they’re overwhelmed … if you’re not transparent from the get-go, it’s really hard to rebuild the trust.” (Case 3 –Interview–Customer Success) “Typically, for myself the contracts … is clear and explicit list of expectations so that we have something to fall back on and say, this is where we need added support … so that we can make sure that we are having our needs met, and providing you with enough information so that you can meet those needs.” (Case 3 –Interview–Data Engineer)
***Risk Assessment*** *Do risk assessments*, *plan pre-emptively*, *create systemic accountability*	- Tooling to keep track of risks (e.g., risk register) - Clear communication and escalation mechanisms - Awareness of regulations to abide by - Patience, empathy, and other people skills - Social learning	- Lengthy processes - Lack of transparency with partner organization - Limited time and resources - Uncertain and rapidly changing circumstances that make it difficult to map out the risk space	“So I think for me the big risks are like there’s not enough time to be clear on what you’re doing together so sometimes you get off track there, there’s not enough time to identify the benefits for all parties and have a lot of good clarity around that. And then I also think sometimes you skip over risks that can sometimes become real downstream in the project and you have to deal with them down there rather than dealing with them at the start in an emergency situation.” (Case 1 –Interview–Vice President) “There’s understanding on both ends as long as we’re being very clear about why we can’t answer something or can we answer something in a different way [due to reidentification and privacy risks]. It’s always met with understanding, and the fact that they’re able to ask those questions. . . pushes us to be able to better understand how we can ask questions of the data on an ongoing basis.” (Case 3 –Interview–Data Engineer) “So the contracts that we put in place are like, we only want, like the deidentified, aggregated data." (Case 3 –Interview–Chief Data Officer)
***Development*** *Embrace privacy by design*, *engage in testing*	- Regular touch points to keep abreast of changing needs - Ability to pilot technology / workflow with a small set of users - Patience, empathy, and other people skills - Social learning	- Lack of regular communication / touchpoints - Lack of end user buy-in - Technology / solution not conducive for a pilot - Limited time and resources	“So, we would always have that continuity between that one key person who’s talking both with the data provider works with the data team intensively and is involved with any meetings with the client, whenever it comes to using the data. So just making sure there’s continuity so there’s never that gap in communication I think it’s important, and super helpful.” (Case 3–Interview–Data Scientist) “… we did do this on a very small scale, like a pilot project before we actually did this in a larger way at one home with a very highly motivated group of individuals, and what I did was I presented at their… physician advisory committee meeting, and that meeting occurred once every month, and being able to rapidly obtain key information from them what worked well what did not work well almost in like a PDSA cycle format and being able to implement … was really the key to success here.” (Case 1 –Interview–Clinical Champion)
***Deployment and Evaluation*** *Communicate proactively and continuously*, *keep records and capture learnings*	- Transparency - Mechanisms to learn from progress - Patience, empathy, and other people skills - Social learning	- Lack of alignment on core problem to be solved - Difficulty with measuring outcomes - Limited time and resources	“I think like the most important thing is just ensuring that everyone is on the same page and everyone is working towards the right the right goal. If people are kind of misguided and doing things for the wrong reasons, then the partnership just falls apart.” (Case 1–Interview–Clinical Champion) “We’re really trying to formalize our processes and so now we meet as a team… bi-weekly and one of the standing agenda items is to talk about challenges, successes, key learnings. So we try to establish a culture of information sharing… we do it a bit more informally… rather than having a “playbook” that we update every time.” (Case 1 –Interview–Implementation Manager) “…knowing what you want, being able to have a good open relationship to be able to talk about what’s needed or what works and what doesn’t.” (Case 2 –Interview–Chief Information Officer)

“*So if you’re in a crisis*, *I just don’t know that you have time for that kind of lengthy [procurement] process because*, *there are there are pretty significant consequences for a loss of time and*, *you know*, *time is non-renewable as a resource you don’t get it back—you can’t rewind the clock… And so that’s the question*: *do you want to spend that time in that process*? *I think there needs to be a more agile process*.*” (Case 3—Interview—Chief Executive Officer)*

However, there was a dichotomy to the effect of the pandemic on public-private partnerships: the same time pressures that posed challenges for the partnership also served as a catalyst for them. Participants in the private sector across our three cases felt that the business value of their solutions was made much more apparent during the pandemic. For example, virtual care proliferated in March 2020 because providers needed the means to connect with patients when outpatient clinics were shut down. Moreover, the public sector organizations were able to move much faster during the pandemic to form partnerships partly because leadership had a “burning platform” to act, especially if inaction would also cause harm. Leadership buy-in was an enabler to removing “red tape” that would otherwise stall partnership formation.

“… *that pressure of time*, *it cuts through really debating what’s real and what is like*, *not a real risk … we’ll sometimes spend months debating like an indemnity clause in a contract about like a potential catastrophic event that’s very unlikely to occur… We were able to move past what would have been months and months of discussion… We were able to make a lot of ground in an emergency situation because we felt that any potential risk was outweighed by the risk of losing lives to COVID-19*.*” (Case 1—Interview—Vice President #1)*

### Early and sustained alignment around the problem is core to success

We found that a clear understanding of a central problem was critical to the success of our studied partnerships. Participants noted that successful partnerships were the ones where effort was made by the vendor to deeply understand the client’s pain points during the sales cycle and longitudinally through the partnership.

“*So each party I think really has to… figure out where their little Venn diagrams can overlap in the middle to the success of both… in a pandemic that same approach would be even more profoundly important*, *because speed is not on your side there*. *You gotta move quicker*, *faster*, *and so you better define that common space in the middle*, *much faster than you would in normal times*.*” (Case 1—Interview—Incubator Lead)*

Once a partnership was underway, participants consistently stressed the importance of continuous transparency around motivations, deliverables, progress, and roadblocks. Transparent communications were viewed as a core enabler of trust that both parties were aligned towards solving the same problem.

“…*having that open*, *honest*, *and clear communication will allow just*, *you know for that trust to be built*.*” (Case 2—Interview—Customer Success)*

**Fig 2:**
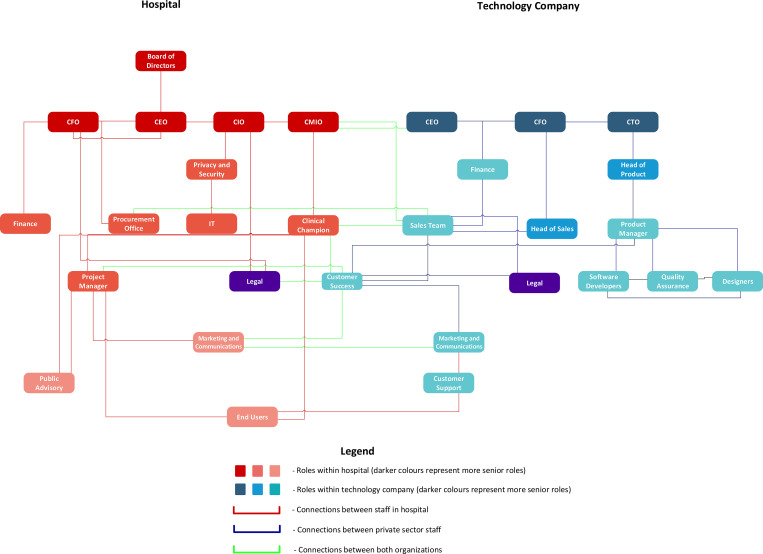
Generalized partnership logic diagram between technology companies and hospitals.

**Fig 3:**
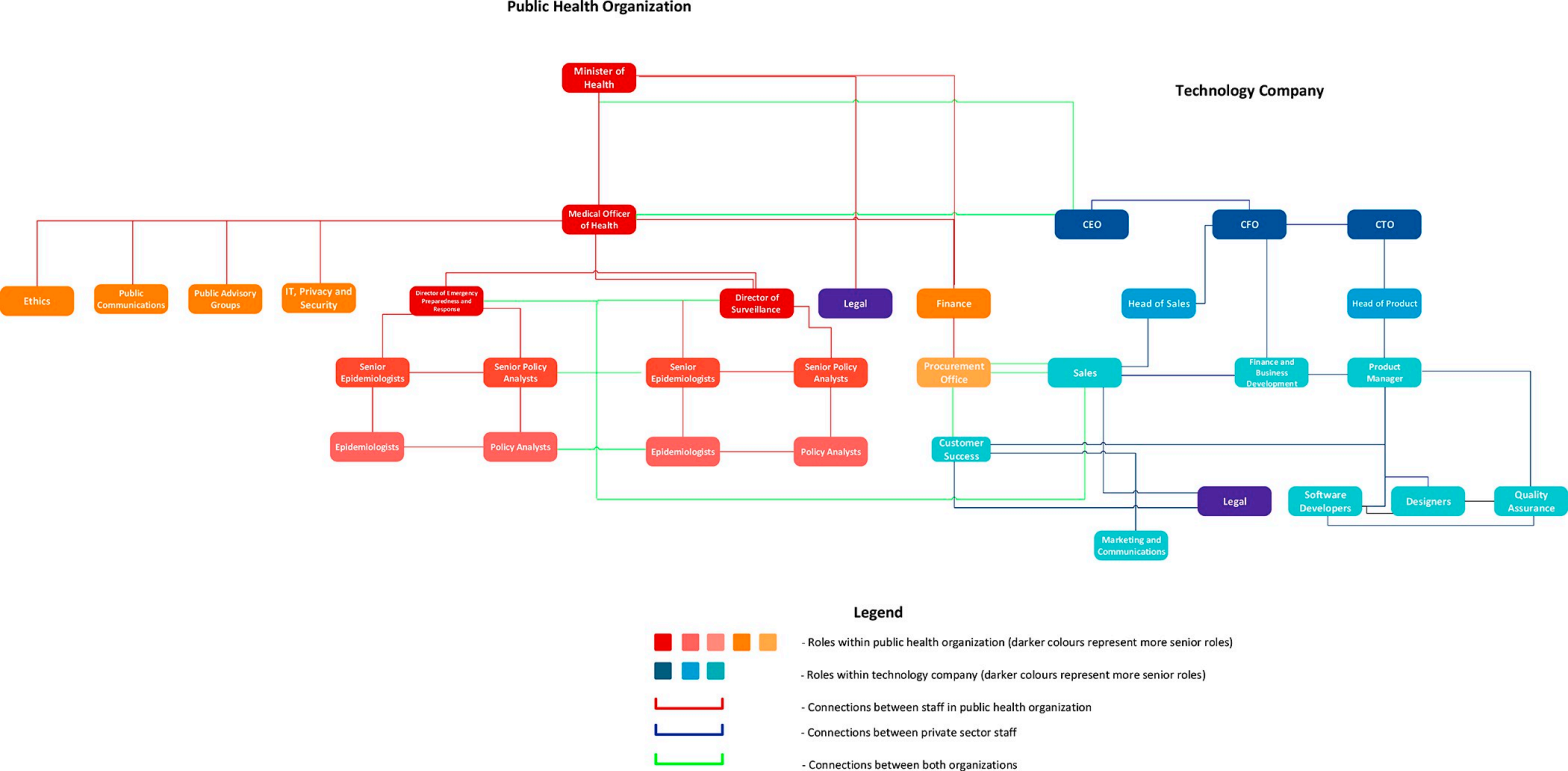
Generalized partnership logic diagram between technology companies and public health organizations.

The success of the partnerships in our case study is also reflected in the establishment of governance structures that support early and continuous alignment through transparency and trust. Logic diagrams demonstrate the multi-level connections between partner organizations, both in healthcare (Fig 2) and in public health (Fig 3). In both cases, the client success role has high centrality within the governance structure and serves as a vital “bridge” between the partners.

“*It’s my role within the company to be the voice of the client so I need to understand what the client wants*, *their needs*, *their priorities and advocate for them within the company… We are basically at the cross-section of almost every single department*, *because we are the voice of the client and the client drives the product*.*” (Case 3—Interview—Customer Success)*

Beyond customer success, participants also spoke to the importance of ensuring alignment between different levels of seniority across the partnership and being able to escalate issues appropriately. One participant called this the “zipper:”

“*I zipper people in and I make them develop relationships… Those contact points determine success or failure… So as an example [if] you were the director level or PM*, *I’d have you and your other person report monthly to [tech company A CEO] and myself… And you live and die together…[you need] the zipper*, *the alignment of interest*, *the processes to cause reporting*, *and the honesty to escalate when something’s going wrong [to] deal with it at the right level*, *you can’t deal with a strategic issue at an operational level*.*” (Case 1—Interview—Chief of Staff)*

### The emergency context led to the triage and streamlining of governance processes

Table 3 shows the wide range of tools that were used to govern the partnerships (i.e., governance processes) across our three cases of study. Participants noted that the time and resource pressures of the pandemic led to shifts in certain governance processes, such as identifying goals and allocating tasks, from formal mechanisms (e.g., project charters) to more informal ones (e.g., rapid, huddle-style meetings).

**Table 3:** Common government processes utilized across our three cases of study.

Governance Processes	Description	Quotes
***Contracts***	- Setting expectations between parties - Mitigates risks - Holds parties accountable for certain deliverables, on certain timelines - Examples include master service agreements, end user license agreements, etc.	“I think every decision should be as evidence as informed as possible. Usually in crisis, you, you feel that that has to go…so don’t sign a ten year contract… put it on a, you know, six month contract with the option of renewal (Case 1-Interview-Chief Medical Information Officer) “We have a master service agreement with [tech company C CEO] and he agreed in our contract … we could extend it to anyone in our Ontario Health Team, and they named all the partners … But then what we did, though, was he did sign separate contracts with each of those groups that were actually coming on to our instance” (Case 2-Interview–Chief Information Officer)
***Documentation***	- Provides records of key decisions/events for parties to look back onto - Supports building institutional knowledge	“We’re using SharePoint now … everything is on SharePoint and we you know we work on communal documents that way as well. But definitely you know documenting methods and having them in an easy to find spot for everyone to access is, is important. . .” (Case 3-Interview-Data Scientist)
***Key Performance Indicators (KPIs) and Metrics***	- Varying processes done to measure progress towards a specific goal - Measuring the success or lack of towards an organization’s objectives in a partnership	“In terms of developing KPIs I think it’s important to look at what the strategic plan of the, you know, hospital or the service or the long-term care sector … It’s important to look at the strategic plan. And what usually within the strategic plan, you’re able to see you know what is important … When you’re able to actually see what the [quality improvement and patient safety goals] are and how those line up with the project … it actually also gets different key stakeholders on the same page.” (Case 1-Interview-Clinical Champion) “There’s going to be stakeholders [who] will… think you can help with reducing burnout and it’s not completely defined because how do you define burnout right so it’s like, I think you can reduce the amount of time that people are taking to reach other people which can reduce burnout but there’s not a set of concrete, measures…” (Case 2 –Interview -CEO)
***Meetings***	- Many different styles and variations; ranges from formal presentations to stakeholder daily operational huddles - Tool to ensure transparency and alignment throughout the partnership - Provide updates between parties - Informally, the “glue” that holds partnership together	“… some of its out of my hand. . . That being said, we’ll always develop as much as possible close relationship to my main contact with a client and we’ll have actually separate meetings just one on one, that are very transparent. Usually, especially if I have a good relationship they can be very blunt, and they’ll sit there and outline, maybe the priorities at the time or the issues at the time. And really what they need from us to do, to deal with it. And that’s exactly what we’ll do in terms of codifying it.” (Case 3-Interview-Client Success)
***Privacy Assessment***	- Formal process done to determine how a program/service could affect the privacy of an individual - Helps avoid the potential negative effects of privacy breach	“Once we got a few hospitals, that’s when you can connect the Privacy Officer at one hospital to another one and be like, oh yeah let’s share notes. Until then, it was a privacy document that we created with our external privacy firm and the credibility was incomplete there … you can always get the hospital do all a privacy impact assessment on you, but if you make them do the entire work, you can probably assume that’s a lot of resources so your sale is probably not gonna go through” (Case 1 –Interview–CEO) “From a modeling point of view. I think for the most part you always want to have, as disaggregated data as possible because it allows you to do a lot more analysis at that level. Obviously, complete disaggregation comes with huge privacy and ethical concerns so that’s… always kind of taken off the table” (Case 3-Interview-Senior Epidemiologist)
***Procurement***	- Formal process of finding and agreeing to terms and acquiring services from external sources - Done through a tendering or competitive bidding process - In Ontario, broader public sector guidelines exist when buying solutions over $100,000	“The fragmented purchasing that we see in Ontario means it’s very very difficult for us to grow here in Canada. . . I can only get procurement from one hospital at a time.. . .It’s a very, very long procurement process. And for me to get to meaningful scale here Ontario I’m gonna have to get a lot of hospitals to buy my products, it’s very hard for me to be a successful company here in Ontario … So, the way that we procure in Ontario certainly drive some of our emerging technology superstars in the healthcare space to go south of the border or to other countries versus to try to operate in our market." (Case 1-Interview-CEO) “I appreciate why there is a broader public sector procurement rules in place, because often, you may miss out on maybe something that actually would achieve your needs better because you’re not actually considering all options, so that’s why there’s this forced procurement which allows for more open bidding and you may actually get better responses right… now again pros and cons to it…” (Case 2-Interview-Chief Information Officer) “Well, I think the key thing in a time of crisis is like time is everything right. . . an RFP process is equitable and it will open it up but there is a period of creating the RFP there’s a period of putting it out there and giving people time to apply going through a review process, etc. So, I mean these are the trade-offs that you got to decide like do you have that time, and like what is the cost of creating a very systematic process when you’re in the middle of a crisis. . ." (Case 3-Interview-CEO)
***Product Roadmap***	- Living document that maps out the strategy, deliverables, and goals for a product overtime - Essential tool for prioritizing development of new features - Aid in achieving alignment between stakeholders in partnership	“I worked with them to kind of say, “Okay, this is a product roadmap that that we think is important for you” to develop a product to meet our needs and I think they saw that that would be generally applicable to other customers in their in their market and so it was again a symbiotic relationship…” (Case 2 –Interview–Chief Medical Information Officer) “There’s like tactical things like keeping [clients] aware of advancements to the product so like any new features that might be coming out to communicating more long-term vision … And also relaying that information back to us, if there is you know specific requests and so on.” (Case 3 –Interview–Product Manager)
***Project Charters***	- Formal document that clearly outlines the project objective - Also includes a project’s scope and responsibilities - The importance of such a document has been met with mixed opinions	“…I believe it’s imperative… it has been my experience that you have to get into some of those details and you have to write them down and you have to look at them a few times and go over them and read them out loud together in order to really make sure that you’re teasing out any assumptions people may be making about what we’re going to achieve together.” (Case 1-Interview-Vice President) “They’re almost traditional artifacts that brought peace of mind to the traditional old guard on how things are done. …you can get by without an official charter as long as you almost have like a, like a charter cheat sheet … like a one pager or some sort of living like mission statement or principles that you’re guiding the implementation … I still think charters are important and they’re ideal if you have the time to build a robust one … but by no means do I think that they’re, they’re mandatory.” (Case 1-Interview-Sales)
***Risk Register***	- Formal document used as a risk management tool - Done to fulfil regulatory compliance for all risks identified - Includes information about each risk, the nature of the risk, and mitigation measures	“… the kind of the risk log or risk register something that’s just regularly reviewed and updated based on how the project is going so you can get ahead of things or mitigate the things as best as you can.” (Case 1-Interview-Sales)
***Security Assessment***	- Formal process to test an organization’s security preparedness - Checks for vulnerabilities within technology systems and business processes - Can include recommendations to lower the risk of future attacks	"I’m talking about cybersecurity. Typically when you do these things you like to do threat risk assessments you like to do penetration tests and things like that. I’m given limited time. You may not always have the luxury of doing the things that you would like to do to ensure you know that level of due diligence. . . so what controls are in place to ensure that, for example, anyone who needs access to the system is access is granted on, you know, least, least privileged required to do their job. . . . ensuring that security has been implemented to align with, you know, hospital policy as an example. . . things like you know password policies is a simple example." (Case 1-Interview-Vice President)
***Stakeholder Engagement***	- A process done to identify the needs of key stakeholders - Includes the identification, analysis, planning, and implementation of actions in which key groups are present	“…community health groups or community groups, organizations they already exist… you don’t have to make up a new body. They already exist. They are already doing work. Go talk to them and it could be as simple as just having a phone call with them. You don’t need to set up like a complicated consultation body and process…you do need to make sure that those voices are embedded throughout which obviously demands some kind of governance… rethinking of those mechanisms, but yeah, they already exist—so use them.” (Case 3 –Interview- Policy Analyst)
***Standards and Certifications***	- Certifying products to meet standards (e.g., ISO) - Helps signal buyers and competitors the adherence to best practice - Determined by independent third-party	“[Tech Company A software] is now listed as validated … for virtual care… and meets all of the provincial privacy, security, interoperability, and technical requirements…” (Case 1-Document) “Security is … evaluated based on security standards … there’s not a healthcare regulation on security. So people [are] evaluated on like different, different ones so one of standards ISO 27001, [ISO] 27002 so evaluated based on that … so we hired a privacy firm and we also hired a security firm… to expedite our sales process.” (Case 2-Interview-CEO)

*“… clearly identifying what the goals are and articulating what the accountabilities are, my sense is that in an emergency this happened, far more informally than someone literally drafting up a charter or terms of reference … [it would] be like, … “All right, we’re gonna have a huddle, we’re going to spend 30 minutes this way, you’re going to do this, we’re going to do this. All right, let’s go””. (Case 3—Interview—Chief Executive Officer)*

While many governance processes were modified to be more efficient, participants also stressed certain processes that were vital and needed to be maintained. For example, stakeholder assessment could not be skipped in its entirety but leveraging the public organization’s ownership and deep understanding of their problem and context allowed for one private organization to identify and prioritize “directly-impacted” groups during an accelerated stakeholder assessment. Moreover, mechanisms around what Blasimme and Vayena term “monitoring” such as maintaining risk registries or keeping track of key performance indicators, could not be dropped altogether because they helped the partnership remain aligned and navigate emerging risks. This is especially true in the context of privacy or security issues.

“*I’d say that getting approval and acknowledgment from stakeholders… is a step you just can’t skip… if the stakeholder groups asked you to do something*, *you should have acknowledgment and awareness at a senior team level that it’s being done*.*”(Case 1—Interview—Chief Security and Privacy Officer)*

An unexpected finding was that participants did not prioritize publicly sharing details of the partnership. Leaders stressed that, especially in the “information overload” context of the pandemic, publicly-directed communications about a given partnership should be meaningful and prioritized around the key needs of stakeholders.

“…*especially in the context of COVID for example*, *[it’s] not always the most appropriate thing to be necessarily like flooding a hospital’s Instagram account to try to market this type of program when like they’re trying to get vaccinations in arms*.*” (Case 1—Interview—Implementation Manager)*

### Social learning can offset some emergency pressures

Social learning, defined as learning through observing others [55], was identified by Blasimme and Vayena as a key enabler of governance by systemic oversight and was identified across all three study cases. For the hospitals, social learning occurred both informally (e.g., Chief Information Officers would reach out to each other for advice) and formally (e.g., the local university’s medical school co-ordinated city-wide COVID-19 response tables for hospital leadership that met as often as daily). For some participants, social learning was viewed as a bare minimum level of due diligence needed before embarking on a partnership with a vendor.

“*As much as a crisis makes people make rapid decisions—don’t [do that]*. *Take the time to at least do a*, *you know*, *an environmental scan and say like*, *“hey*, *what is everybody else doing*?*”” (Case 1—Interview—Chief Medical Information Officer)*

The public health organization we studied also had a small team dedicated to learning from jurisdictions worldwide, but it was less clear how to take these learnings and apply them to their own context compared to the hospitals.

“*I think there was an effort to constantly learn from other countries… there’s a group whose only task is to look at other countries*, *what they’re doing*, *and how they’re performing as a result of what they’re doing… and that project has been ongoing for at least probably since the summer of 2020*.*” (Case 3—Interview—Senior Epidemiologist)*

For the private technology companies, social learning took the form of press releases, when deemed appropriate, to signal key milestones (e.g., launching new products or services). Conversely, it also involved scanning for other offerings in the market to understand and crystallize their unique value proposition as a company. For one partnership, they noted that social learning was formalized by the nature of their membership in an innovation accelerator, where they delivered and heard presentations about successful and unsuccessful healthtech partnerships from other participants in the program.

### Startups have a vital role to play in emergency response, but growth can introduce new challenges

Since the companies studied were startups, we found that they were highly attuned to their local contexts and could often be highly flexible and adaptable to create bespoke solutions for their partners. Several participants from the hospitals contrasted their previous experiences with major electronic medical record (EMR) companies based in the United States, positively reflecting on how their perspectives as end-users were much more valued and regularly informed product updates compared to the EMR companies.

“*I think with the big vendors*, *the other thing is making sure that I’m not getting the short end of the stick where I’m paying a lot but actually not getting service… whereas I think those discussions are easier with a smaller company right because they just have more flexibility” (Case 2—Interview—Chief Information Officer)*

All three startups studied scaled dramatically (i.e., doubled, tripled) during the pandemic due to the increased demand for their solutions. While on the one hand, the increased demand and revenue is positive and reinforce their value, aspects of pandemic-fueled “hypergrowth” were also challenging. For example, one dimension of growth was around staffing and building capacity to complete core tasks in response to increased demand, such as responding to customer support tickets or onboarding new users.

“*[Virtual care company] experienced a 500% increase in usage and added over 200 healthcare providers to its platform in one month alone*.*” (Case 1—Marketing brochure)*

Such growth often demands changes to governance processes to improve their quality or feasibility.

“*So for example… deployments*, *before it was just [the] wild west*, *we have this hospital deploying whatever and we think about things off the top of our head… and mistakes happen… With our new process… when we have a hospital deployment*, *we have every single task created… okay these are things that we have to do*, *we can delete ones that are not applicable and that makes it much more repeatable and the quality is much better on a wider scale”*.*—(Case 2—Interview—Chief Executive Officer)*

Given the urgent, and oftentimes bespoke, nature of client demands during the pandemic, the companies we studied also had to navigate how to maintain their core value proposition.

“*Each client wants a slightly different version of what you’re trying to offer*. *And so you start to get this just like infinite list of things that you can build against but it only applies to one client at a time*, *and building like bespoke things at scale is just not possible*.*” (Case 3—Interview—Product Manager)*

### There’s no partnership without healthy, motivated teams

The intensity of the pandemic timelines and workloads universally created burnout and a high degree of turnover among teams in both the public and private sectors. Effective interpersonal skills and emotional intelligence, especially empathy, was viewed as an enabler of effective partnerships.

“*I’ll typically relate back to you know I get it*, *I’m a healthcare professional myself*, *you know*, *things come up*, *emergencies come up… I think that that perspective allows me to be there in a more supportive way because I do understand that their environment can be very hectic and can be ever-changing*.*” (Case 2—Interview—Customer Support)*

Participants also spoke to the need for visibility into governance processes. Leaders felt the onus to be active throughout the partnership and diagnose areas of potential misalignment quickly.

“*I understood that in that forming stage*, *it required a tremendous amount of effort and time*. *And the stakes were high*. *So the reality is… I’d be talking to that CEO*, *three times a day*. *And I’m setting up a steering committee with the executives there*. *And I’d be monitoring it… And [I would] be there when it’s going wrong*. *People like to be there when it’s going right*.*” (Case 1—Interview–Chief of Staff)*

Having key governance processes, such as discussions on ethical issues or company direction, being highly visible and inclusive helped to keep teams motivated and engaged, especially in virtual and thus potentially isolating or siloed work environments.

“*It really speaks to you know being in that supportive environment where we can listen to everyone’s concerns…So the fact that we are able to bake in*, *you know*, *being considerate of a client’s time and everyone’s time but still take the due diligence in order to be able to say can we [navigate this challenge] differently and more responsibly*, *I’m very happy to be a part of something like that*. *(Case 3—Interview—Data Engineering)*

Ultimately, one of the best remedies against burnout and promoters of effective team functioning was that participants on both the public and private side of the partnerships we studied found that their work during the pandemic was valuable and impactful.

“*Working in healthcare as a non-clinician… you want to be able to draw that line between what you’re doing and what the patient impact is in as short a distance as possible*. *And I think what we’ve been able to do during the pandemic is as short a distance between those two points as you can get*.*” (Case 1—Interview—Sales)*

## Discussion

We found that key aspects of adaptive governance were adopted in three distinct partnerships between health organizations and private technology companies to deploy digital technologies as part of COVID-19 pandemic response. While the emergency context created considerable pressures with respect to time and resources, the partnerships we studied were able to use a wide range of governance structures and processes to ensure they were continuously aligned on shared goals. We found that governance processes were continuously refined and streamlined in response to logistical pressures but that certain processes, such as stakeholder engagement and monitoring for the emergence of new risks, were identified as essential. Most of the insights were drawn from key informant interviews, as publicly available documents did not frequently address the inner workings of these partnerships. Taken together, our findings suggest that effective and responsible cross-sector partnerships to deploy digital technologies during health emergencies are possible with several elements that enabled successful collaboration outlined in our findings. However, some learnings can be used to design and improve future health technology (healthtech) partnerships.

Both Blasimme and Vayena’s framework of adaptive governance through systematic oversight and Gasser *et al*.’s navigation aid were helpful when applied to our real-world cases [20,45–47]. Although Blasimme and Vayena’s framework was designed specifically around big data research, we found that many concepts were applicable to digital technologies more broadly. For example, Blasimme and Vayena defined “reflexivity” as an awareness of the harmful, classificatory impact of machine learning algorithms, but we found reflexivity to be applicable in broader terms such as an awareness of how deploying virtual care technology could cause harm due to the digital divide [56–58]. Our key informants were able to enact many aspects of these two frameworks into practice, even though they were not explicitly used or perhaps were not aware of them. Thus, an important area for knowledge translation could be to improve the visibility of these frameworks to key actors engaging in partnerships via integration in formal education or upskilling. Participants often spoke to best practices from their respective fields as guiding their work, such as plan-do-study-act (PDSA) cycles for clinicians and the Information Technology Infrastructure Library and The Open Group Dependability Through Assuredness^TM^ (O-DA) Architecture Framework for informaticians [59–61].

While frameworks and best practices can be helpful while governing private sector partnerships, our findings also emphasize that firms should not necessarily chase a “silver bullet” governance arrangement. Rather these higher-level guidelines on governance structures and mechanisms should be interpreted as such. Across our cases of study, we found a high degree of “bricolage”, or improvisation, by actors in both the private and public sector to navigate their partnership [62,63]. Given the importance of experimentation, it stands to reason that partnerships that are in place prior to an emergency can leverage the “peacetime” environment to conduct the necessary experimentation to determine what works best in their own context. Experimentation around governance models has long been explored in the realm of climate governance, but is relatively new in digital health [64].

Our findings also demonstrate the importance of viewing digital technology deployments in health as complex, socio-technical systems. It would be incorrect to assume that private sector actors can “parachute in” and fix problems, especially in a crisis. As digital technologies change workflows, teamwork across a wide range of stakeholders is needed to repair these workflows and move forward to a new equilibrium [65]. The success of the partnerships in our study hinged on meaningful engagement across the entire pipeline from problem identification to solution deployment and evaluation. Health organizations should focus on their understanding, and ultimately ownership, of the core problem to ensure alignment for the partnership. Marwaha and colleagues highlight the role of key players within the health system such as clinical champions and executive sponsors as drivers of this alignment, but our work also demonstrates that it becomes important who they are connected to on their partner side and how [66]. At a broader level, human resource and capital considerations (e.g., burnout, people skills) were not captured in our guiding frameworks but should not be ignored as a key driver of effective and responsible governance [67].

The healthtech landscape in industry is largely bimodal, with major, incumbent multinationals (e.g., Google, Apple, Microsoft) on one side and startups on the other [41]. A key strength of our work was to focus on the relatively understudied role of smaller companies and startups in the context of a public health emergency. Our findings support existing literature that startups are highly agile and able to tailor their offerings to local needs, which makes them valuable contributors to crisis response [35]. However, the pandemic has created an additional layer of stress on firms that already have to navigate uncertain business conditions and reveals areas of improvement to better support these vulnerable entities. Healthtech startups must be careful to maintain their core value proposition and grow sustainably, even when an emergency context provides an impetus for new partnerships to form with hospitals and public health organizations. In many provinces in Canada, hospitals and public health organizations are public entities that are subject to public sector accountability acts. These acts outline rules for procuring technologies that are meant to promote fairness, competition, and the responsible spending of taxpayer dollars. However, many respondents felt that these guidelines make the procurement process too slow, cumbersome, and stifle the ability of non-incumbents to compete given lengthy sales cycles—even outside of an emergency context. To address these issues, the United Kingdom created a Procurement Policy Note in March 2020 that outlined criteria for public sector purchasers to issue direct awards or launch competitions with accelerated timescales [68]. Outside of emergencies, innovation accelerators embedded in health organizations can offer a testbed for governance experimentation, increase risk tolerance, and formalize social learning. The National Health Service (NHS) has developed a series of pathways to foster the invention, development, and implementation of innovations, including digital technologies [69,70]. These pathways exist to, among other functions, accelerate otherwise labyrinthine procurement processes for small and medium-sized companies, create pilot sites for deployments in partnership with academic medical centers, and support a class of fellows in scaling innovations across the country [69,70]. Similar accelerators exist at academic medical centers in the United States, such as at the University of California San Francisco and Mass General Brigham [71,72].

The interpretation of our findings should be considered in light of certain limitations. Firstly, the three cases of study in this work do not exhaustively cover every application of pandemic technology but are broadly representative of major categories (i.e., clinical, operational, and epidemiological). Secondly, the partnerships studied here can be deemed successful (i.e., they all reached the deployment phase) and maintain ongoing business relationships. Thus it is possible there is an element of social desirability as well as recall bias among our key informants leading to an overly positive appraisal of the partnership and events therein. Throughout our interviews, we attempted to address this issue and obtain critical perspectives by probing participants on what they thought could have gone differently or be improved as well as reassuring them that we would protect their anonymity (i.e., both personally and organizationally) to help them speak freely. Most interviews were conducted with managers and other participants at senior levels within the organizations of study, but we believe these perspectives are appropriate since it is leadership who generally steer the direction and priorities of partnerships. Finally, our study relied on publicly available documents—which we found were helpful for outlining that a partnership formed or key outcomes but not often why certain decisions were made and how those outcomes were achieved. This is in contrast to a popular study by Powles and Hodgson who obtained documents (e.g., contracts) on the partnership between the NHS Royal Free Trust and Deepmind through a freedom of information request [22]. We addressed this limitation in publicly available documents by conducting extensive interviews with key informants. These interviews were contingent on goodwill and building trust with our key informants by conveying that our study sought to explore how to ultimately improve the functioning of future partnerships. A freedom of information request can quickly undermine this goodwill. We caution that researchers consider the benefits and drawbacks of such an approach.

The limitation on publicly available documents also speaks to an important area of future work. When asked about transparency, our key informants stressed that publicly-directed communications about a given partnership might not be appropriate because communication should be meaningful and prioritized around key stakeholder needs. This finding creates tension with the prevailing view in the literature that transparency is essential for maintaining social license [11,73,74]. Indeed, there has been criticism of public registries of artificial intelligence algorithms in Amsterdam and Helsinki since they are not, in isolation, a sufficient governance solution to ensure ethical use [75]. Unfortunately, we were unable to provide guidance around how those communications, and to a further extent public engagement, should be operationalized. Standards, regulations, and expectations around transparency are also highly likely to be variable between jurisdictions. Future work should explore how to effectively and pragmatically foster transparency and public engagement around partnerships between health organizations and technology companies, especially in the context of a health emergency. Finally, we believe that as partnerships between digital health organizations and technology companies increase in frequency, future work should continue to take conceptual frameworks and seek to explore their relevance and impact empirically in real-world contexts.

## Conclusion

Strong public-private partnerships are essential to maintain resilient health systems. Despite the considerable time and resource pressures of the COVID-19 pandemic, we found that key aspects of adaptive governance, such as social learning and flexibility around governance processes, helped foster effective and responsible partnerships between health organizations and technology companies. COVID-19 has accelerated the digital transformation of health systems globally, and a return to the pre-pandemic normal is increasingly unlikely. Stakeholders must leverage the learnings from the ongoing pandemic to navigate not only future crises but also as private technology companies continue to expand their role in health.

## Supporting information

S1 File Appendix A–Anonymized Search Terms. Appendix B–Consent Form. Appendix C–Semi-structured Interview Guide. Appendix D–Coding Guide.
(PDF)
