# Supplementary material for: Governing partnerships with technology companies as part of the COVID-19 response in Canada: A qualitative case study
Source: PLOS Digit Health. 2022 Dec 14;1(12):e0000164. doi: 10.1371/journal.pdig.0000164 (PMC9931354; doi:10.1371/journal.pdig.0000164)
Supplement: S1 File — Appendix A–Anonymized Search Terms. Appendix B–Consent Form. Appendix C–Semi-structured Interview Guide. Appendix D–Coding Guide. (PDF) [file pdig.0000164.s001.pdf]

Governing Partnerships with Technology Companies as Part of the COVID-19 Response in Canada: A Qualitative Case Study

Supplementary Material

Contents:

Appendix A – Anonymized Search Terms
Appendix B – Consent Form
Appendix C – Semi-structured Interview Guide
Appendix D – Coding Guide

Appendix A – Anonymized Public Document Search Terms

Square brackets indicate substitution with the company's name or the individual's name. For example, if company A was named Stark Industries and the company's CEO was named Tony Stark, the corresponding Google search would be "[Stark Industries] [Tony Stark]".

We manually clicked through every page in the websites of the three technology companies. However, for the health organizations, we searched for the partner company. For example, if hospital B was named Ontario General Hospital, we would search for "[Stark Industries]" in Ontario General Hospital's website's search bar.

Partnership	Anonymized Search Terms
Case 1 - Clinical<br>• Technology Company A (A)<br>• Hospital B (B)	• [A]<br>○ Manual search of all sub-pages<br>• [A] [A CEO name]<br>• [A] [A COO name]<br>• [A] [A CTO name]<br>• [B]<br>○ Search for [A]<br>• [A] "and" [B]<br>• [A] "and" [B] "and" COVID-19<br>• [A] telemedicine COVID-19
Case 2 - Operational<br>• Technology Company C (C)<br>• Hospital D (D)	• [C]<br>○ Manual search of all sub-pages<br>• [C] [C CEO name]<br>• [C] [C COO name]<br>• [C] [C CTO name]<br>• [D]<br>○ Search for [C]<br>• [C] "and" [D]<br>• [C] "and" [D] "and" COVID-19<br>• [C] COVID-19
Case 3 - Epidemiological<br>• Technology company E (E)<br>• Public Health Organization F (F)	• [E]<br>○ Manual search of all sub-pages<br>• [E] [E CEO name]<br>• [E] [E COO name]<br>• [F]<br>○ Search for [E]<br>• [E] "and" [F]<br>• [E] "and" [F] "and" COVID-19<br>• [E] COVID-19

Appendix B – Consent Form

[NAME] [DATE]
[ORGANIZATION]

Operationalizing Effective and Responsible Governance in Partnerships with Private Technology Companies as Part of the COVID-19 Response in Canada – A Qualitative Study

Dear [NAME],

You have been invited to take part in a research project entitled "Operationalizing Effective and Responsible Governance in Partnerships with Private Technology Companies as Part of the COVID-19 Response in Canada – A Qualitative Study".

It is entirely up to you to decide whether to take part in this research. If you choose not to take part in the research or if you decide to withdraw from the research once it has started, there will be no negative consequences for you, now or in the future.

Introduction:
Medical and public health organizations have sought to leverage digital technologies to bolster their pandemic response. Partnerships with private technology companies, both large and small, have become commonplace in order to design and deploy these technologies. The goal of this study is to gain an in-depth understanding of the barriers and enablers of responsible collaborations between public healthcare organizations and private technology companies in Canada to design and deploy technological solutions as part of the response to the COVID-19 pandemic.

Purpose of study:
The purpose of this project is to conduct key informant interviews with employees involved in partnerships between medical/public health organizations and private technology companies during COVID-19. Specifically, this study will: identify how healthcare organizations and private technology companies considered and navigated the barriers and enablers of collaborating during a pandemic.

What you will do in this study:
You will participate in a semi-structured interview over Zoom. The interview will take up to an hour. Specifically, you will be asked about your experience in the partnership across the phases of: preparation, planning, risk assessment, development, and deployment and evaluation. Please note that no personal or organizational information will be collected for our study purposes and all responses will be de-identified.

Possible Benefits:
This study will identify key barriers and enablers of collaborations between health organizations and private technology companies. This work is important because it will ultimately provide the basis for more effective collaborations across sectors to promote resiliency to emergencies. This will serve as an important contribution for stakeholders to improve their emergency response.

Possible Risks:
Since no personal or organizational identifying information will be collected or disclosed at any time, the potential for social risks during or after the interview is low. There is a potential risk of managers or supervisors asking if you have participated in the study, however we have requested that they do not do so and that all participants are not obligated to provide this disclosure. There is a potential that some questions will make you feel uncomfortable, however you are free to decline answering those questions.

Confidentiality:
Interviews will be recorded and transcribed for analysis purposes. The transcribed copies of the interview, raw data, notes, and digital records will be kept in a password protected digital folder and only two members of the study team will have access to them. All digital recordings will be destroyed once transcribed.

While direct quotes will not be presented with the name or organization of the individual who provided the quote, they will be presented with the role (e.g. product manager) in the final publication/presentation. It is still possible that the nature of the response makes them identifiable. However, since interview content will be pooled across multiple individuals and organizations the risk is low and all efforts will be made to minimize this risk.

Anonymity:
No personal information will be collected in the course of this research. You will not be identified in any reports or publications without explicit permission.

Reporting of results:
Study results will be published in a scholarly journal and/or presented at a conference. The investigators will protect your identity in all publications and presentations.

Questions:
You are welcome to ask questions at any time during your participation in this research. If you would like more information about this study, please contact Vinyas Harish at [email] or [phone number].

The ethics protocol for this research has been reviewed by the Health Sciences Research Ethics Board at the University of Toronto (#26448). If you have ethical concerns about the research (such as the way you are being treated or your rights as a participant), you may contact [name] (Research Ethics Manager, Health Sciences) at [email] or [phone number].

Consent:
Your signature on this form means that:
- You have read the information about the research
- You have been able to ask questions about this study
- You understand what the study is about and what you will be doing
- You understand that you are free to withdraw from the study at any time, without having to give a reason, and that doing so will not affect you now or in the future
- You have the ability to skip questions you do not wish to answer
- You are under no requirement to report whether or not you participated to your managers

If you sign this form, you do not give up your legal rights, and do not release the researchers from their professional responsibilities.

You will be given a copy of this form for your records.

Signatures:

Participant

1. I have read and understood the description provided; I have had an opportunity to ask questions and my questions have been answered. I consent to participate in the research project, understanding that I may withdraw consent at any time.

Your Signature: ____

Date (mm/dd/yyyy) : ________________________

2. I agree that the interview will be electronically recorded and transcribed.
   _____ Audio only recording
   _____Audio and video recording

   Note: All digital recordings will be destroyed once transcribed.

Your Signature: ____

Date (mm/dd/yyyy) : ________________________

Researcher:

I have explained this study to the best of my ability. I invited questions and gave answers. I believe that the participant fully understands what is involved in being in the study, any potential risks of the study, and that they have freely chosen to be in the study.

Researcher Signature: ____

Date (mm/dd/yyyy) : ________________________

Appendix C – Semi-structured Interview Guide

Preamble to be read verbatim:
The purpose of this project is to conduct key informant interviews with employees involved in partnerships between medical/public health organizations and private technology companies during COVID-19. Specifically, this study will: identify how healthcare organizations and private technology companies considered and navigated the ethical challenges of the solutions in question along the pipeline of their development to deployment, compare the barriers and enablers of responsible governance, and determine the specific contextual influences and pressures brought on by the emergency nature of the pandemic.

Your willingness to proceed with the interview means that:

- You have read the information provided about the research
- You have been able to ask questions about this study
- You understand what the study is about and what you will be doing
- You understand that you are free to withdraw from the study at any time, without having to give a reason, and that doing so will not affect you now or in the future
- You have the ability to skip questions you do not wish to answer
- You are under no requirement to report whether or not you participated to your managers

As a reminder, you are not giving up your legal rights, and do not release the researchers from their professional responsibilities.

From your consent form, you stated that you [do OR do not] agree to being recorded (audio), recorded (video), and transcribed. Is that still the case?

[If consent is provided for recording]
I have now activated the recording function on Zoom.

[If consent is provided for live transcription]
I have now turned on the live transcription feature in Zoom. You should now be able to see text appear at the bottom of your screen in real time. If you find this distracting you can hide this text by clicking the arrow beside the “Live Transcript” button at the bottom of the Zoom window and then clicking “Hide subtitle.”

Shall we begin with the interview?

1 hr interview

Screening Question:

1. Reflecting on your experiences, what is the role of the private sector in responding to health emergencies?

Phase 1: Preparation

2. Based on your experience, how do partnerships with the public/private sector form? How do leaders gauge if a partnership is appropriate? *(Prompt: Personal connections?*)

3. Who do you perceive to be the key players needed to guide a successful partnership between private technology companies and a health organization? (*Prompt: Public representative?*)

4. Do you feel that there are any guiding principles that are important to establish at the start of a partnership? *(Prompt: Time of the essence, making things open)*

Phase 2: Planning

5. How important is it to establish clear objectives with your partner organization? (*Prompt: Is it always possible to define what success looks like?*)

Phase 3: Risk Assessment

6. What are the possible risks that can arise in partnerships between private technology companies and health organizations? (*Prompt: Plan for known and unknown risks? Short and long term? Considerations on data? Non-expiration?*)

7. Reflecting on your experience, what mechanisms are helpful to mitigate these risks and ensure accountability? (*Prompt: Privacy/bias/ethics impact assessment? Data usage agreement? Non-disclosure agreement?)*

Phase 4: Development

8. What sorts of safeguards are important to build into digital solutions as they are being developed? (*Prompt: Technical? Legal? Organisational? Privacy by design/default? Disclaimers, security in terms of access, safeguards for whom?)*

9. How do partnerships navigate testing and validating a solution before it is deployed? Is it clear how much testing is appropriate? (*Prompt: Role of third-party audit?*)

Phase 5: Deployment and Evaluation

10. How important is ongoing communication between parties once digital solutions are deployed? What makes communication effective?

11. Do you think there is a role for broader public communication strategies when partnering with the private sector?

12. How important is it for teams to engage in real-time monitoring and feedback when a digital solution is deployed? (*Prompt: How to navigate the emergence of new risks "in the real world?"*)

13. How important is it for teams to keep track of records and learnings?

Overall:

14. Who benefits most from partnerships between private technology companies and health organizations, and why? (*Prompt: the public?)*

15. Overall, when considering partnerships between private technology companies and health organizations, what is needed to make them work effectively? What makes partnerships difficult? *(Prompt: Mechanisms to overcome barriers?)*

16. What sorts of challenges do health emergencies add to effectively partnering with the public/private sector? What becomes easier? (*Prompt: Navigating uncertainty?)*

17. If you were to give advice to leaders about to navigate a partnership with the public/private sector, what is the most important thing you would tell them?

18. Is there any additional information or comments you'd like to share?

19. Can you recommend anyone else I should talk to get a different view on this?

30 minute interview:

1. Do you feel that there are any guiding principles that are important to establish at the start of a partnership?

2. What are the possible risks that can arise in partnerships between private technology companies and health organizations? (*Prompt: Plan for known and unknown risks? Short and long term? Considerations on data? Non-expiration?*)

3. Reflecting on your experience, what mechanisms are helpful to mitigate these risks and ensure accountability? (*Prompt: Privacy/bias/ethics impact assessment? Data usage agreement? Non-disclosure agreement?)*

4. How do partnerships navigate testing and validating a solution before it is deployed? Is it clear how much testing is appropriate? (*Prompt: Role of third-party audit?*)

5. How important is it for teams to engage in real-time monitoring and feedback when a digital solution is deployed? (*Prompt: How to navigate the emergence of new risks "in the real world?"*)

6. Overall, when considering partnerships between private technology companies and health organizations, what is needed to make them work effectively? What makes partnerships difficult? *(Prompt: Mechanisms to overcome barriers?)*

7. What sorts of challenges do health emergencies add to effectively partnering with the public/private sector? What becomes easier? *(Prompt: Navigating uncertainty?)*

8. Do you think there is a role for broader public communication strategies when partnering with the private sector?

9. If you were to give advice to leaders about to navigate a partnership with the public/private sector, what is the most important thing you would tell them?

10. Can you recommend anyone else I should talk to get a different view on this?

Appendix D – Original Coding Guide

The results from the transcribed interviews will be described using a coding structure that includes (but will not be limited to) the following domains:

1. **Position**
    a. Current position
    b. Role in partnership

2. **Adaptivity**
    a. Proper management of novel data streams

3. **Flexibility**
    a. Treat different data types based on use and not just source

4. **Inclusiveness**
    a. Who were the stakeholders involved, who was missing?

5. **Responsiveness**
    a. To emerging risks, both short and long term
    b. To shifting context

6. **Reflexivity**
    a. Understand and mitigating disparate impact on marginalized groups from classificatory activities

7. **Monitoring**
    a. Ongoing risk awareness/assessment to anticipate emergence of new vulnerabilities and/or undesirable outcomes

8. **Governance structures:**
    a. Centrality
    b. Density
    c. Cohesion

9. **Governance processes**
    a. Social learning
    b. Complementarity
    c. Visibility
